# Supplementary material for: A Likelihood Approach for Real-Time Calibration of Stochastic Compartmental Epidemic Models
Source: PLoS Comput Biol. 2017 Jan 17;13(1):e1005257. doi: 10.1371/journal.pcbi.1005257 (PMC5240920; doi:10.1371/journal.pcbi.1005257)
Supplement: S1 File — (TAR.GZ) [file pcbi.1005257.s014.tar.gz › HSPH_Online-SI-Revision/IPoi09/n1-severe/n1-severe_BENCH_table-8.pdf]

1	R0	{1.51081, 13.7078}	{1.34133, 4.115}	{{1.25474, 3.11158}, {1.454, 5.65591}}	{11.8931, 69.9806}	inf	20.	25.6207	{0., 51.}	80.1724	0.516524	newly	8.	38.7586	{0., 75.}	411.207	0.813032	Reff	1.3839	0.898421	{0.691801, 1.15882}	35.0806	ar	656.	450.724	{290., 560.}	31.2921
2	R0	{1.73182, 11.3873}	{1.71767, 8.56908}	{{1.22516, 3.26445}, {2.47514, 15.904}}	{24.4337, 44.6143}	inf	10.	79.9048	{9., 190.}	713.333	0.820399	newly	5.	53.4762	{16., 93.}	980.	0.970897	Reff	1.66774	1.54611	{0.898908, 2.17666}	25.3944	ar	653.	740.119	{503., 910.}	26.8614
3	R0	{1.50449, 10.3474}	{1.18026, 2.80992}	{{1.12171, 1.78492}, {1.24719, 3.82585}}	{21.5508, 72.8442}	inf	24.	13.7778	{2., 28.}	51.8519	0.504808	newly	17.	25.4444	{2., 58.}	102.614	0.477116	Reff	1.41873	0.943697	{0.726051, 1.14701}	33.4831	ar	651.	282.667	{69., 464.}	56.5796
4	R0	{1.63493, 9.1955}	{2.05914, 13.1738}	{{1.35478, 6.00596}, {2.65407, 19.3194}}	{33.0925, 55.0409}	inf	6.	7.92537	{1., 19.}	96.2687	0.414351	newly	4.	5.68657	{1., 17.}	111.567	0.489754	Reff	1.61368	2.07897	{1.42607, 2.57266}	33.9387	ar	663.	658.836	{6., 907.}	39.1009
5	R0	{1.53822, 8.05553}	{1.74348, 13.1616}	{{1.25721, 7.7153}, {2.26161, 19.1478}}	{23.1058, 69.2696}	inf	7.	9.70909	{0., 24.}	110.909	0.608337	newly	6.	7.29091	{0., 16.}	110.606	0.709669	Reff	1.51515	1.77473	{1.33229, 2.35158}	23.4478	ar	621.	538.745	{3., 874.}	42.3364
6	R0	{1.69374, 17.0793}	{1.58058, 15.069}	{{1.27716, 8.76637}, {1.81374, 19.2972}}	{11.3753, 18.1545}	inf	15.	16.8182	{2., 45.}	74.2424	0.393694	newly	6.	9.47727	{2., 22.}	102.652	0.362592	Reff	1.64801	1.54581	{1.26047, 1.77161}	11.2087	ar	662.	574.886	{292., 745.}	20.8356
7	R0	{1.42179, 11.8031}	{1.81804, 13.481}	{{1.39507, 7.40677}, {2.22832, 18.788}}	{29.5621, 31.9151}	inf	25.	44.5185	{4., 97.}	125.778	0.464011	newly	16.	25.6667	{2., 55.}	109.722	0.444708	Reff	1.34786	1.64769	{1.17406, 1.99427}	28.3669	ar	517.	707.833	{445., 837.}	46.5685
8	R0	{1.46683, 6.95875}	{2.16146, 13.1476}	{{1.3618, 6.0993}, {2.84915, 18.4275}}	{51.724, 98.3889}	inf	9.	11.6066	{1., 26.}	101.093	0.463092	newly	10.	8.80328	{1., 21.}	91.3115	0.612898	Reff	1.44043	2.21258	{1.75026, 2.7601}	55.1024	ar	653.	702.311	{7., 914.}	41.7393
9	R0	{1.34525, 11.505}	{2.04337, 15.8945}	{{1.68954, 11.6019}, {2.30371, 19.5128}}	{51.895, 41.1783}	inf	14.	48.193	{6., 100.}	266.04	0.578671	newly	10.	23.8596	{3., 49.}	169.123	0.524557	Reff	1.2874	1.87203	{1.60544, 2.16183}	46.0009	ar	532.	770.561	{690., 880.}	54.7751
10	R0	{1.77958, 14.372}	{2.18556, 16.7474}	{{1.81391, 11.799}, {2.46533, 19.5287}}	{23.7689, 22.916}	inf	20.	42.9697	{5., 90.}	148.333	0.442609	newly	5.	21.6364	{3., 47.}	351.515	0.607526	Reff	1.68348	2.01904	{1.67931, 2.28884}	22.3849	ar	671.	797.409	{730., 892.}	27.8801
11	R0	{1.60334, 19.9548}	{1.81887, 15.4196}	{{1.55644, 10.5556}, {1.99771, 19.2013}}	{14.656, 22.7276}	inf	26.	58.6429	{13., 120.}	148.077	0.407025	newly	11.	28.9643	{6., 58.}	181.494	0.442057	Reff	1.48149	1.52326	{1.1354, 1.87568}	15.6273	ar	617.	712.304	{589., 825.}	23.3474
12	R0	{1.68623, 7.58585}	{1.92583, 12.9514}	{{1.15964, 8.35044}, {2.688, 18.33}}	{29.0686, 74.2184}	inf	4.	4.61972	{1., 10.}	84.507	0.444972	newly	7.	3.46479	{0., 7.}	73.4406	0.761928	Reff	1.67443	1.93509	{1.2015, 2.67032}	28.8751	ar	640.	492.155	{1., 920.}	56.5119
13	R0	{1.82689, 18.0022}	{1.80171, 14.0405}	{{1.48482, 9.08517}, {2.19504, 19.0355}}	{12.038, 23.961}	inf	23.	36.2745	{5., 81.}	103.922	0.435525	newly	6.	20.7451	{3., 42.}	271.242	0.580034	Reff	1.74285	1.68042	{1.34205, 1.97574}	11.8821	ar	673.	673.961	{523., 830.}	19.535
14	R0	{1.75911, 9.16883}	{1.56336, 14.4438}	{{1.05515, 8.34284}, {2.44675, 18.834}}	{27.3023, 61.5868}	inf	4.	3.58904	{0., 9.}	85.6164	0.57087	newly	3.	1.93151	{0., 6.}	83.105	0.783757	Reff	1.75031	1.58218	{1.06559, 2.48267}	26.3004	ar	661.	346.466	{1., 880.}	62.6718
15	R0	{1.46168, 11.3859}	{1.85034, 11.4569}	{{1.40191, 5.65591}, {2.37462, 18.743}}	{28.0287, 29.871}	inf	25.	95.08	{28., 155.}	284.8	0.519748	newly	14.	56.38	{24., 86.}	304.143	0.558672	Reff	1.32428	1.35446	{0.829931, 1.92608}	23.1453	ar	556.	737.1	{575., 870.}	34.8813
16	R0	{1.53577, 11.7581}	{2.11924, 12.2424}	{{1.34436, 5.4396}, {2.80569, 17.5123}}	{43.1196, 32.2423}	inf	5.	13.8857	{0., 38.}	237.143	0.667073	newly	2.	11.8286	{0., 31.}	544.286	0.781261	Reff	1.52041	2.14049	{1.41302, 2.73782}	44.1301	ar	625.	658.457	{2., 926.}	47.0766
17	R0	{1.70698, 9.72924}	{2.07699, 10.4319}	{{1.31023, 4.12341}, {2.74056, 16.1449}}	{31.4139, 37.8204}	inf	8.	6.97059	{1., 16.}	68.75	0.494144	newly	5.	8.76471	{0., 19.}	148.824	0.641082	Reff	1.68138	2.14761	{1.39093, 2.80008}	33.4366	ar	675.	632.235	{3., 920.}	41.8998
18	R0	{1.5973, 14.931}	{1.84477, 14.1415}	{{1.17434, 8.35044}, {2.53075, 19.3211}}	{28.6958, 22.4985}	inf	9.	3.59211	{0., 9.}	68.8596	0.734893	newly	7.	2.60526	{0., 7.}	71.0526	0.886792	Reff	1.57973	1.86413	{1.18463, 2.51237}	29.1844	ar	632.	483.618	{1., 901.}	55.2319
19	R0	{1.87468, 17.004}	{1.45765, 7.87767}	{{1.28596, 5.27199}, {1.67961, 11.6019}}	{22.6351, 53.9238}	inf	36.	44.3171	{13., 68.}	59.5528	0.343339	newly	15.	38.8537	{10., 65.}	177.886	0.522119	Reff	1.69659	1.1358	{0.807669, 1.40099}	34.1414	ar	692.	551.171	{435., 697.}	22.1979
20	R0	{1.84715, 12.531}	{1.90715, 16.2544}	{{1.66328, 12.378}, {2.11476, 19.5406}}	{9.08612, 31.531}	inf	31.	46.8333	{8., 99.}	91.2903	0.361437	newly	22.	21.9167	{4., 39.}	54.3182	0.343872	Reff	1.71785	1.74108	{1.47336, 1.97282}	9.47595	ar	661.	753.017	{633., 849.}	18.762
21	R0	{1.75938, 13.5959}	{2.14656, 7.60832}	{{1.31023, 3.04996}, {2.92375, 11.7575}}	{35.5729, 44.5412}	inf	12.	68.2787	{0., 158.}	503.689	0.906809	newly	10.	76.377	{0., 168.}	698.197	1.02087	Reff	1.71891	1.89922	{1.11648, 2.55651}	29.0472	ar	620.	702.213	{7., 936.}	47.2475
22	R0	{1.63456, 10.7469}	{1.61731, 12.8603}	{{1.25721, 7.80621}, {1.98971, 19.2013}}	{12.746, 35.5762}	inf	9.	29.4524	{3., 70.}	258.466	0.546403	newly	9.	18.4524	{2., 38.}	148.413	0.507964	Reff	1.57572	1.54017	{1.1624, 1.92324}	17.2344	ar	654.	609.429	{372., 813.}	22.5426
23	R0	{1.62702, 19.1392}	{1.4457, 4.8518}	{{1.30094, 3.11158}, {1.67405, 7.28757}}	{16.6439, 74.65}	inf	40.	40.1714	{8., 79.}	52.4286	0.352812	newly	13.	50.5714	{9., 95.}	304.396	0.649093	Reff	1.4692	1.16309	{0.758821, 1.5437}	28.7863	ar	600.	526.314	{410., 678.}	21.4048
24	R0	{1.70466, 12.1202}	{1.95659, 11.2832}	{{1.70777, 8.49961}, {2.2143, 14.4309}}	{15.4448, 18.3607}	inf	32.	125.661	{52., 191.}	297.824	0.601853	newly	23.	71.9286	{36., 103.}	218.634	0.517575	Reff	1.43191	1.10869	{0.606258, 1.68278}	31.753	ar	613.	784.696	{718., 871.}	31.4583
25	R0	{1.61735, 11.3169}	{1.37064, 11.9557}	{{1.18406, 6.74711}, {1.56048, 16.8028}}	{16.111, 31.5358}	inf	25.	23.8519	{2., 65.}	77.7778	0.565419	newly	15.	12.6667	{2., 35.}	68.3951	0.510007	Reff	1.53163	1.31104	{1.02228, 1.55576}	15.9246	ar	681.	453.778	{37., 647.}	33.366
26	R0	{1.56977, 12.3035}	{1.76413, 13.6539}	{{1.20997, 6.77341}, {2.35866, 18.9086}}	{23.7771, 30.7841}	inf	16.	14.3571	{0., 38.}	89.0625	0.784135	newly	8.	9.58929	{0., 28.}	111.384	0.786655	Reff	1.53367	1.74798	{1.20029, 2.1602}	21.7999	ar	594.	530.643	{4., 845.}	47.3545
27	R0	{1.46317, 5.8298}	{2.48983, 13.8918}	{{1.89254, 8.41615}, {2.8833, 17.2484}}	{70.2131, 139.092}	inf	8.	49.7536	{7., 101.}	529.529	0.696208	newly	8.	34.8116	{6., 79.}	344.928	0.566008	Reff	1.39294	2.29375	{1.76433, 2.71695}	64.897	ar	586.	868.275	{735., 936.}	51.0486
28	R0	{1.70002, 14.9806}	{1.58257, 13.8923}	{{1.0804, 8.42022}, {2.2143, 18.5502}}	{23.9003, 21.5091}	inf	4.	3.88406	{0., 11.}	89.1304	0.642728	newly	0.1	2.3913	{0., 7.}	2369.57	0.876003	Reff	1.68812	1.62187	{1.09913, 2.23516}	24.016	ar	652.	352.449	{1., 853.}	61.263
29	R0	{1.52729, 16.1014}	{1.81639, 14.9039}	{{1.53473, 10.5289}, {2.036, 19.2116}}	{19.6797, 17.556}	inf	40.	76.8246	{13., 150.}	123.289	0.379694	newly	22.	37.	{10., 64.}	95.1356	0.319241	Reff	1.40358	1.42091	{1.04116, 1.86569}	17.4021	ar	592.	719.175	{601., 823.}	26.5677
30	R0	{1.72795, 13.5635}	{1.39769, 5.40447}	{{1.28596, 3.98513}, {1.454, 7.40677}}	{19.662, 60.3184}	inf	42.	30.8333	{2., 61.}	58.4921	0.516722	newly	22.	35.7667	{2., 73.}	106.212	0.480551	Reff	1.5396	1.00036	{0.754859, 1.27574}	35.0249	ar	668.	461.367	{131., 632.}	31.0529
31	R0	{1.51591, 18.1544}	{1.83056, 12.7288}	{{1.38815, 6.77341}, {2.26161, 19.1478}}	{24.2484, 31.6277}	inf	41.	73.9038	{13., 147.}	115.525	0.393198	newly	22.	42.1731	{11., 76.}	119.493	0.384367	Reff	1.4007	1.43141	{0.919533, 1.9144}	22.0283	ar	541.	705.25	{512., 876.}	36.7873
32	R0	{1.4282, 10.3534}	{1.84705, 13.8994}	{{1.32705, 7.47062}, {2.27287, 19.1478}}	{32.6292, 45.8108}	inf	4.	15.434	{2., 30.}	303.774	0.538431	newly	3.	9.56604	{1., 19.}	245.283	0.503888	Reff	1.40535	1.8944	{1.42619, 2.31464}	36.089	ar	521.	691.66	{37., 881.}	54.4997
33	R0	{1.56693, 11.3972}	{2.04645, 11.1515}	{{1.24271, 4.25615}, {2.76795, 18.2464}}	{39.6645, 33.7436}	inf	11.	6.35385	{1., 16.}	70.2098	0.642449	newly	6.	6.47692	{0., 14.}	103.333	0.667371	Reff	1.54029	2.13955	{1.3956, 2.76241}	44.3081	ar	695.	634.646	{3., 928.}	40.8921
34	R0	{1.50675, 19.8823}	{1.72502, 9.33176}	{{1.58886, 7.29751}, {1.93231, 12.4507}}	{14.7364, 53.065}	inf	62.	82.7273	{37., 131.}	54.1056	0.197212	newly	30.	57.7273	{28., 87.}	98.6364	0.280697	Reff	1.22047	1.01451	{0.661173, 1.40727}	25.1846	ar	587.	697.182	{589., 785.}	19.6376
35	R0	{1.72889, 7.76068}	{2.24191, 9.59967}	{{1.43579, 4.47101}, {2.80569, 14.1724}}	{36.2073, 40.2931}	inf	6.	9.12698	{1., 23.}	111.905	0.468475	newly	7.	11.254	{0., 27.}	132.88	0.597325	Reff	1.70469	2.29783	{1.57287, 2.82441}	38.0266	ar	650.	721.952	{3., 911.}	41.9023
36	R0	{1.62644, 4.12303}	{2.00387, 10.7854}	{{1.1315, 4.47101}, {2.74988, 18.2815}}	{37.0334, 163.219}	inf	5.	2.84	{0., 8.}	85.6	0.834881	newly	11.	5.27	{0., 12.}	101.182	1.28621	Reff	1.61343	1.99395	{1.13037, 2.7336}	37.2184	ar	652.	407.33	{1., 921.}	65.8482
37	R0	{1.70878, 15.8072}	{1.68129, 12.3246}	{{1.42446, 7.81552}, {1.8951, 16.0085}}	{8.61298, 24.2853}	inf	53.	72.8367	{25., 125.}	61.9946	0.238907	newly	18.	42.3878	{15., 70.}	145.692	0.384118	Reff	1.48835	1.21794	{0.885525, 1.52636}	20.2045	ar	674.	666.49	{519., 788.}	10.4463
38	R0	{1.58967, 6.80568}	{2.31426, 11.1668}	{{1.54465, 5.30296}, {2.88523, 15.0981}}	{48.5547, 73.1951}	inf	6.	17.6061	{2., 49.}	226.768	0.528764	newly	10.	16.4697	{1., 41.}	121.97	0.52687	Reff	1.55947	2.34566	{1.77559, 2.82175}	51.5609	ar	610.	800.121	{527., 930.}	44.7789
39	R0	{1.344, 8.19498}	{1.64964, 10.7153}	{{1.27231, 5.40945}, {2.12588, 16.748}}	{25.286, 51.4723}	inf	10.	25.1087	{3., 49.}	184.565	0.495911	newly	8.	20.6522	{2., 46.}	200.	0.529382	Reff	1.2889	1.59988	{1.14099, 2.03022}	30.0892	ar	551.	602.261	{79., 842.}	37.6785
40	R0	{1.61509, 7.90825}	{1.95081, 12.2686}	{{1.14983, 6.31117}, {2.87797, 17.9732}}	{36.9573, 62.4896}	inf	9.	1.93	{0., 5.}	79.8889	1.0649	newly	10.	1.72	{0., 4.}	87.4	1.34677	Reff	1.59894	1.94508	{1.14868, 2.87509}	37.3138	ar	581.	346.03	{1., 917.}	75.1205
41	R0	{1.60017, 5.90605}	{2.19455, 14.2223}	{{1.6082, 8.77064}, {2.76849, 18.9406}}	{39.9637, 142.827}	inf	8.	16.0469	{1., 38.}	148.242	0.487848	newly	8.	11.4688	{1., 29.}	116.406	0.545121	Reff	1.56817	2.18817	{1.62573, 2.75329}	41.3484	ar	661.	753.187	{402., 924.}	35.3064
42	R0	{1.64436, 15.1337}	{2.08761, 9.53125}	{{1.31023, 3.26445}, {2.7943, 15.3124}}	{37.2469, 39.1375}	inf	21.	62.2679	{9., 129.}	219.983	0.540287	newly	9.	51.9107	{4., 112.}	495.833	0.77207	Reff	1.5605	1.97065	{1.22063, 2.54225}	35.6818	ar	640.	778.768	{385., 939.}	38.005
43	R0	{1.48917, 11.917}	{1.70086, 16.5893}	{{1.54363, 13.1646}, {1.8189, 19.6311}}	{14.6516, 39.3184}	inf	31.	41.	{7., 86.}	76.6749	0.338073	newly	19.	19.25	{3., 40.}	61.8421	0.381774	Reff	1.38791	1.53131	{1.30073, 1.76905}	14.0913	ar	551.	667.673	{559., 759.}	26.0889
44	R0	{1.65577, 7.89317}	{2.19829, 13.0996}	{{1.41571, 6.71734}, {2.78517, 18.2127}}	{38.8438, 74.6746}	inf	6.	23.0968	{2., 54.}	314.516	0.589199	newly	9.	16.1613	{1., 38.}	133.692	0.507906	Reff	1.63093	2.25268	{1.64572, 2.66884}	40.5709	ar	576.	780.887	{530., 931.}	52.2989
45	R0	{1.6909, 12.3275}	{2.04457, 12.1058}	{{1.42659, 5.7404}, {2.59602, 17.005}}	{25.4523, 24.6105}	inf	23.	45.8	{9., 87.}	129.644	0.38042	newly	14.	30.7273	{3., 63.}	150.649	0.429464	Reff	1.5793	1.97472	{1.52749, 2.61117}	28.3274	ar	675.	811.364	{712., 918.}	22.9764
46	R0	{1.62351, 16.0698}	{1.71458, 12.7267}	{{1.32613, 6.25107}, {2.11476, 18.765}}	{15.0941, 26.6815}	inf	17.	41.8103	{5., 90.}	178.6	0.521592	newly	10.	24.7931	{3., 54.}	177.241	0.485886	Reff	1.55532	1.59388	{1.21869, 1.95702}	16.0667	ar	591.	654.983	{490., 833.}	29.5379
47	R0	{1.52372, 4.38599}	{2.00387, 10.7854}	{{1.1315, 4.47101}, {2.74988, 18.2815}}	{42.0022, 148.27}	inf	3.	2.84	{0., 8.}	103.333	0.70427	newly	3.	5.27	{0., 12.}	194.333	0.929187	Reff	1.51458	1.99395	{1.13037, 2.7336}	42.0678	ar	618.	407.33	{1., 921.}	68.4838
48	R0	{1.44306, 11.788}	{2.28929, 15.7252}	{{1.94282, 11.3733}, {2.64373, 19.7855}}	{58.6413, 34.8888}	inf	30.	84.6087	{14., 154.}	200.386	0.463985	newly	21.	45.1739	{11., 81.}	136.508	0.404508	Reff	1.32617	1.90111	{1.52014, 2.4059}	43.927	ar	534.	845.725	{759., 914.}	58.3754
49	R0	{1.63624, 11.3893}	{2.19349, 12.063}	{{1.29927, 4.21286}, {2.78517, 17.9172}}	{41.6328, 32.9456}	inf	10.	20.4844	{0., 51.}	165.156	0.65811	newly	9.	18.25	{0., 58.}	167.361	0.686174	Reff	1.60188	2.16616	{1.32673, 2.74911}	41.1597	ar	643.	673.516	{5., 924.}	43.6066
50	R0	{1.46113, 11.1322}	{1.76128, 12.7784}	{{1.21937, 6.16288}, {2.24732, 18.743}}	{26.7279, 33.7665}	inf	12.	25.5962	{2., 59.}	159.776	0.577217	newly	6.	15.5962	{1., 34.}	197.115	0.575923	Reff	1.4246	1.75063	{1.24656, 2.23932}	27.7726	ar	501.	614.596	{27., 863.}	54.9248
